# Ligature of the Common Carotid for Hemorrhage, Consequent on the Removal of a Maxillary Tumor

**Published:** 1872-12

**Authors:** 


### ARTICLE VII.

### *Ligature of the Common Carotid for Hemorrhage, Consequent on the Removal of a Maxillary Tumor.*

H. R., aged 57, gardener, was admitted on May 29, 1872, into St. Thomas' Hospital, under the care of Mr. Le Gros Clark. The most noticeable symptoms in the history of the tumor were rapidity of growth and increase of pain. The pain, which was the first symptom, was not felt until three months before admission, and swelling of the hard palate and upper jaw were noticed a month afterwards. No other member of the family, so far as could be ascertained, had suffered from cancer.

On admission, the patient had a considerable tumor of the upper jaw, distorting the face and forcing its way into the mouth. The eye was partially closed, and there was a discharge from the left nostril, where a polypus was visible. The teeth had disappeared from the left superior maxilla, with the exception of the two incisors. No glandular enlargement of the neck was detected.

On introducing the finger into the mouth, the tumor was felt as a firm elastic mass, and scattered about it were some openings like pinholes. The patient's symptoms were such as might be expected from the character and position of the growth; severe shooting pain in the left side of the head, difficulty of swallowing, much thickening of the voice, and impairment of vision from the upward pressure of the floor of the orbit, so that objects above a certain elevation appeared double. A small portion of the interior of the growth was obtained by harpooning from one of the openings before referred to in the hard palate, and was found to consist of a fibrous nucleated stroma with cells, chiefly of an epithelial character; there were occasional myeloid forms, but a large proportion were very erratic.

On June 12, Mr. Le Gros Clark removed the left superior maxilla. The line of incision passed downward from the inner angle of the orbit by the side of the nose round the left ala nasi to the middle of the lip, and outward along the lower margin of the orbit to the middle of the zygoma The soft growth which was found filling the bone extended so far back that it was impracticable entirely to remove it. Vessels in the tumor under the masseter, bled freely, and were twisted. The actual cautery was applied to the exposed surface, and the superior maxillary nerve cut short. A sponge, covered with gutta-percha tissue, was introduced to fill out the cheek, and the edges of the wound were accucurately adapted with wire sutures.

June 14th.—The sponge was easily removed. The wound was cleaned with Condy's fluid and replugged with oiled lint.

June 17th.—The wound was syringed with Condy's fluid. The incision had healed throughout almost the entire length. All sutures were removed. 7 P. M.—There was copious hemorrhage—about twenty ounces. The bleeding was temporarily arrested by the application of ice and repeated pluggings of lint, dipped in perchloride of iron. The condition of the patient, however, rendered it necessary that there should be some better security against hemorrhage, and accordingly, at half-past ten o'clock, Mr. Wagstaffe tied the common carotid artery. An incision, between two and three inches in length was made over the edge of the sterno mastoid muscle, the skin and fascia were divided, and the superficial veins twisted. The artery, which was large, emerged from under the sterno-mastoid, at about a level with the upper border of the thyroid. A double catgut ligature was used and cut short. The wound was brought together with strips of plaster. The plugs were then removed from the mouth.

June 18th, 6 P. M.—The pulse was small, and the face much blanched. The patient's general condition was somewhat better than was expected. Since the operation the patient had slept a little, and had taken custard milk and iced brandy and water with some relish.

June 20th, 6 P. M.—He had been very restless, and had taken but little nourishment.

June 21st, 6 P. M.—The patient remained in the same state until noon to-day, after which he sank rather rapidly and died at half-past five o'clock. There was no noticeable return of pulsation in the temporal artery. A *post mortem* examination was made by Dr. Payne about twenty-one hours after death. A mass of new growth or inflammatory material remained attached to the under surface of the floor of the orbit, and a small abscess was found under the zygomatic process. Besides this, there was no pus. The left side of the neck was also examined, and the vein and nerves (pneumogastric and descendens noni) were found uninjured. There was very little pus also in this wound. The upper lobes of both lungs were found studded with grouped patches of gray tubercle, and some masses of fibroid tissue, but no caseous matter. The lower lobes contained also scattered gray tubercle. The bronchi were inflamed, and the bronchial glands were large, hyperæmic and succulent, but not caseous. The liver, spleen and kidneys were healthy. The brain also was healthy. The arteries of the circle of Willis contained the ordinary *post-mortem* clots, and a similar clot was found in the upper part of the internal carotid.—*British Medical Journal.*

